# Nanoscale defect evaluation framework combining real-time transmission electron microscopy and integrated machine learning-particle filter estimation

**DOI:** 10.1038/s41598-022-13878-8

**Published:** 2022-06-22

**Authors:** K. Sasaki, M. Muramatsu, K. Hirayama, K. Endo, M. Murayama

**Affiliations:** 1 Department of Science for Open and Environmental Systems, Graduate School of Keio University, 3-14-1, Hiyoshi, Kohoku-ku, Kanagawa, 233-8522 Japan; 2 Department of Materials Science and Engineering, Virginia Tech, Blacksburg, VA 24061 USA; 3 Institute for Materials Chemistry and Engineering, Kyushu University, Kasuga, Fukuoka 816-8580 Japan

**Keywords:** Structural materials, Information technology

## Abstract

Observation of dynamic processes by transmission electron microscopy (TEM) is an attractive technique to experimentally analyze materials’ nanoscale phenomena and understand the microstructure-properties relationships in nanoscale. Even if spatial and temporal resolutions of real-time TEM increase significantly, it is still difficult to say that the researchers quantitatively evaluate the dynamic behavior of defects. Images in TEM video are a two-dimensional projection of three-dimensional space phenomena, thus missing information must be existed that makes image’s uniquely accurate interpretation challenging. Therefore, even though they are still a clustering high-dimensional data and can be compressed to two-dimensional, conventional statistical methods for analyzing images may not be powerful enough to track nanoscale behavior by removing various artifacts associated with experiment; and automated and unbiased processing tools for such big-data are becoming mission-critical to discover knowledge about unforeseen behavior. We have developed a method to quantitative image analysis framework to resolve these problems, in which machine learning and particle filter estimation are uniquely combined. The quantitative and automated measurement of the dislocation velocity in an Fe-31Mn-3Al-3Si autunitic steel subjected to the tensile deformation was performed to validate the framework, and an intermittent motion of the dislocations was quantitatively analyzed. The framework is successfully classifying, identifying and tracking nanoscale objects; these are not able to be accurately implemented by the conventional mean-path based analysis.

## Introduction

In recent years, researchers have been trying to implement machine learning (ML) based approaches in a wide range of scientific fields, and it has attracted considerable attention^1^. ML has demonstrated its capability to implement semantic segmentation, which classifies objects in an image pixel by pixel, and has been applied to practical applications for example, automated driving technology and the medical field^2–7^.

An emerging application of ML is analytical methods for extracting characteristic information about the structure, composition, and properties of various materials, especially nanoscale materials^8–17^. Many reported cases of extracting specific features from a dataset by ML have shown that ML can be further advanced toward a major unbiased data-driven analysis method to gain new insights from the extracted features^2^. This is true also for the temporal series of dataset such as observations of dynamic phenomena, there have also been attempts to use ML-based approaches. Several recent studies employed ML to predict the plastic deformation of micron-scale crystalline solids using data from discrete dislocations dynamics simulations^18^, or to predict microstructure evolution and associated properties changes from spatiotemporal data^19^. In the case of research focusing on crystal defects, ML has been mostly used to classify and semantically segment crystal defects in materials in electron microscope images^8,9,20–22^. Therefore, there are very few examples of ML analyzing the temporal change of crystal defects such as dislocation dynamics directly from experimental observation videos.

The general relationship between the macroscopic deformation behavior of metallic materials and the average dislocation velocity is a well-known fact^23^. However, the quantitative evaluation of dislocation motion has not been studied in details. Dislocations generally exhibit intermittent motions, indicating that it is important to quantitatively evaluate their temporal velocities instead of their average velocities, whereas it has been difficult to capture the motion of individual dislocation and assess their temporal velocities experimentally using transmission electron microscopy (TEM) in the past. Recent dramatic advances in digital imaging technology have made it possible to provide the moment of a nanoscale reaction on a regular laboratory scale^24^. Nonetheless, frameworks for quantitatively analyzing dynamic observation data taken by TEM and linking them to mechanical properties are still in the trial-and-error stage. There are multiple technical challenges for implementing automatic tracking to nanoscale objects such as dislocations in TEM videos even the nanoscale objects are clearly observed in the video due to the TEM's unique contrast generation mechanisms and the nature of nanoscale microstructural features. First, the nanoscale objects of interest must be recognized in a large number of frames in the TEM video data, in which misleading microstructural or image features frequently coexist. Second, the number of valid video frames is almost always smaller than the number of data required for training in ML, making it difficult to universally satisfy the conditions necessary for ML optimization. Third, even after the nanoscale objects of interest are detected, individual objects must be separately tracked throughout the TEM video with being distinguished from others. Especially in the case that the objects repeat unexpected behaviors such as sudden move-and-stop and irregular change of own shape, tracking the objects becomes highly challenging. The unexpected behaviors are often caused by atomic to nanoscale local environment, which is closely related the inhomogeneity of material. Thus, developing a model to predict such behaviors for data analysis would be nearly impossible.

In this study, we developed a ML-based framework for quantitative analysis of nanoscale objects’ dynamic behavior based on the information obtained by detecting the objects in a video using machine learning and tracking the detected objects with particle filters. We confirmed that if a video presents a single experiment, the number of data is sufficient for machine learning to detect dislocations in that video. We then applied the developed ML-based framework to a video in which the dislocation gliding under applied external tensile stresses in a metal was observed using TEM. By detecting and tracking dislocations in the TEM video singly and as a whole using the framework, we were able to calculate the time history of dislocation velocity and quantitatively analyzed its behavior. In particular, we employed the particle filter to the quantitative analysis part of the framework. Thanks to the probabilistic prediction of the particle filter, we successfully captured the unexpected behaviors of individual dislocations.

## Results

When a metallic material is plastically deformed by applying the stress $\tau$, a slip deformation occurs along a specific crystal direction (slip direction) on certain crystal planes (slip plane). Slip deformation is localized by the movement of dislocations, indicated by the "⊥" symbol, on the slip plane as schematically shown in Supplemental Fig. 1^25^﻿.

**Figure 1:**
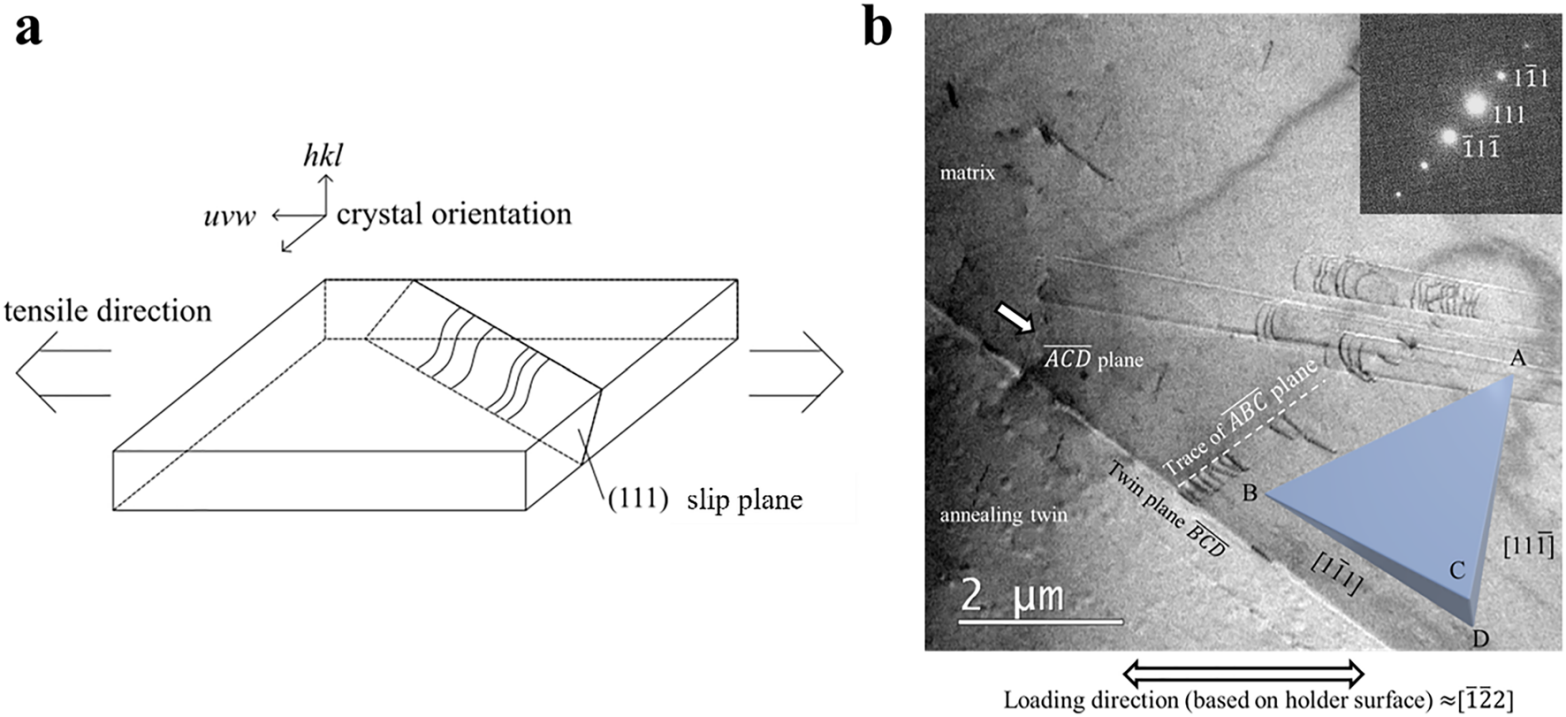
Schematics of in-situ TEM deformation (tensile) experiement. (**a**) Conceptual diagram of the tensile test geometry indicating possible displacements in the geometry of dislocations between projection images and the real space. (**b**) A representive TEM image showing the observed area and its crystallographic orientation.

Assuming no dislocation formation or annihilation occurs, the macroscopic shear strain of a crystal $\gamma$ is expressed as $\gamma = \rho bx$ using the dislocation density $\rho$, the magnitude of the Bugers vector $b$ and the average travel distance of dislocations $x$. Differentiating both sides by time $t$, the strain rate of the crystal $\dot{\gamma }$ can be written by the average migration velocity of the dislocations $v$.

1 $$\dot{\gamma } = \rho bv$$

For the dislocation velocity measurement by TEM observation, Johnston et al. reported one of the first successful cases that measured the average dislocation velocity^23^. They measured the average velocity of the dislocations by dividing the displacement of the dislocations by the time that the stress was applied. However, since the actual dislocation motion is intermittent, a continuous velocity measurement providing the chronological changes is necessary to understand the intrinsic dislocation behavior. Therefore, the overreaching goal of the framework development is to assess the traverse speed of nanoscale objects such as dislocations without compromising the original data’s temporal and spatial resolutions. In this study, we attempt to archive the10 nm/s order temporal and spatial resolutions by applying a U-Net based ML and particle filter integrated method to in situ TEM deformation videos.

The actual validation of the framework proposed in this study was implemented by the following steps described in the rest of this section. The experimental data, TEM videos, were taken during in-situ TEM deformation experiments, in which a high-manganese austenitic steel (Fe-31Mn-3Al-3Si) was subjected to a forced displacement with a tensile rate of 100 nm/s. as shown in Fig. 1a. In Fig. 1b, a group of dislocation lines like arcs moved to the left. Since TEM images represent a 2D projection of a 3D object, the real space geometry of dislocations in the crystalline grain needs to be retrieved to evaluate the stress condition in the observed area. The crystal orientation of the material in the movie is shown in Fig. 1b. In this particular case, the dislocations observed in the movie are moving on the ABC plane and the incident electron beam is transmitted in the direction of $\overrightarrow {{{\text{CD}}}}$ in Fig. 1b. Table 1 summarizes the Schmid factors for the ABC and ABD planes, which indicate the contribution fraction of the load stress to the resolved shear force acting on the slip system.

**Table 1:** Schmid factor.

Loading direction	Slip plane	Slip direction	Schmid factor
[$\overline{1}\overline{2}2$]	ABC	[$1\overline{1}0$]	0.045
		[$\overline{1}01$]	0.136
		[$01\overline{1}$]	0
	ABD	[$1\overline{1}0$]	0.045
		[$\overline{1}01$]	0
		[$01\overline{1}$]	0.045

At the first glance, the position of the field of view (FOV) of the TEM video was not still in order to track down moving dislocations, i.e., there was a large shift of recorded area in the middle of the video. Without fixing the FOV, the velocity of dislocations cannot be measured accurately. We fixed the FOV by i) choose small recognizable features that were in a dislocation-free region and stationary between at least two consecutive scenes and track down the chosen points throughout the TEM video, ii) shift corrections were calculated for the points between scenes , then iii) the corrections were applied to the video under the estimation that the movement of the points were corresponding to that of the scenes. Since the position of each of frames shift relative each other by applying the correct, overlapping parts were cropped throughout the corrected video to finish the FOV fixing.

It should be noted that the optical flow estimation^26–28^ was employed to choose the points in the above step i) and to calculate the displacement by tracking the characteristic points in the step ii). Here, optical flow is a method to track feature points in a video, and commonly used in the computer vision field. As nothing moved excepting the dislocations in the TEM experiment video of this study, optical flow is one of the most suitable methods to fix the FOV of the video.

**Figure 2:**
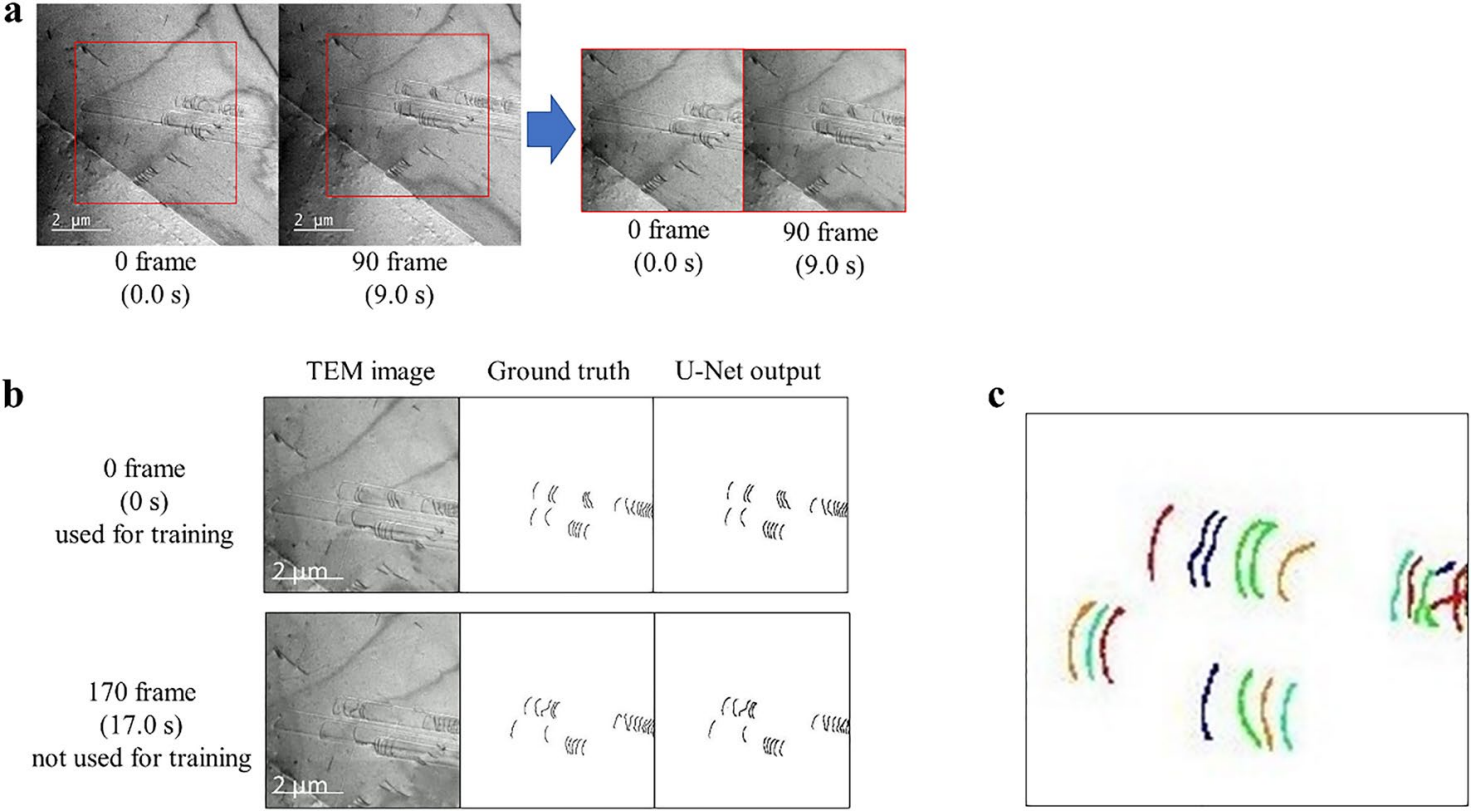
Selected frames from the in-situ TEM deformation experiment video demonstrating the image processing protocol. (**a**) Fixation of the FOV by optical flow: the unmatched coordinates of the FOV were in the left two images (before processing), were aligned by this process. For the following analysis, the FOV was kept consistent for the entire video by applying this process to all frames. (**b**) Binarization of dislocation and background by U-Net. U-Net outputs the same image as ground truth for frames not used for training. (**c**) Identification of each dislocation by image processing shown in Fig. 7. The dislocations were classified into several colors so that neighboring dislocations had different colors. It can be seen that a single continuous dislocation pixel is classified into the same color.

An example of the fixed FOV is shown in Fig. 2a. The cropped region is denoted by the red frame in Fig. 2a, and its size is 320 × 320 pixels. After optical flow process, the FOV does not move within the cropped region. The fixation of the position of the FOV of the TEM video by this process makes it feasible to evaluate dislocation velocities.

In the next step, we performed the semantic segmentation for all frames of the TEM video by ML. Classification of the pixels into dislocation and non-dislocation pixels was conducted on a pixel-by-pixel basis. This mechanical classification makes it possible to objectively evaluate the motion of dislocations among the frames. There are many lines (liner features) in the TEM videos that are not actual dislocations. It would be very time-consuming and tedious to manually detect dislocations in every frame of the video by separating them from other liner feature, as there were dozens of dislocations in one image.

There are two advantages to use ML for this task. The first one is that the detection process is efficient and objective. ML detects dislocations in every frame of the video after learning from a training data which is a correct image set created by the operators. The detection of dislocations in the video will be conducted on the same criteria as the one of the correct image set. The second one is that ML is more robust than numerical filtering. ML is able to detect dislocations without being misled by non-dislocation lines in a TEM image. For these reasons, we thought it is the best to use a ML method to detect dislocations in the video.

There are several ML methods for semantic segmentation, such as FCN^29^ and SegNet^30^. Due to its high performance, U-Net^6^ has been used in many researches^31,32^, therefore we employed U-Net in this study. U-Net is a method developed by Ronneberger et al. that has been succeeded in semantic segmentation for cells in microscope images. We manually traced the first 170 images, i.e., from 1 to 170 frames of the TEM video, to generate 170 correct images. Then we trained U-Net using 1—100 frames as training data and 101—170 frames as test data. Figure 2b shows the output from U-Net. We were able to obtain the same output as the correct image for the test data.

In the last step, in order to track down the same dislocation in the video, we used a particle filter, which is one of object tracking methods in videos. Other methods such as optical flow are commonly used for the object tracking. Optical flow, however, cannot track dislocations accurately. Optical flow cannot track the point which moves quickly and it is difficult to specify the feature points in a line with shape changes, although movements of dislocations may be unpredictable and the shapes of dislocations may change. In this study, we thought that a particle filter approach^33,34^ is more suitable for tracking dislocations. Since particle filter tracks objects using probability distributions, it can retake and keep tracking individual dislocations even if the exact location of more than one dislocations was temporary lost due to a sudden and unforeseen movement. Particle filter is a better fit for this case as the dislocations' shape change likely occur and the movement of dislocations may be unpredictable.

For the use of particle filter, it is necessary to identify individual dislocations in each of video frames. We adopted a method to identify dislocations based on the spatial continuity of pixels belonging to the dislocations. Figure 2c shows an example of how the automatic identification of individual dislocations based on the spatial continuity of pixels of the dislocations worked. The dislocations were colored into different colors based on the ID of them. Fifteen dislocations were identified in this trial by the particle filter, whereas 23 dislocations were visually identified in Fig. 2c. This difference is due to the fact that the current identification protocol requires a sufficient distance between adjacent dislocations to identify them accurately. When several dislocations were closely spaced, two dislocations could be identified as one dislocation especially if they were noticeably bowed. On the other hand, dislocations were properly identified when they were far apart in the entire movie and the dislocations were relatively straight. This study succeeded in tracking dislocations that meet these conditions.

**Figure 3:**
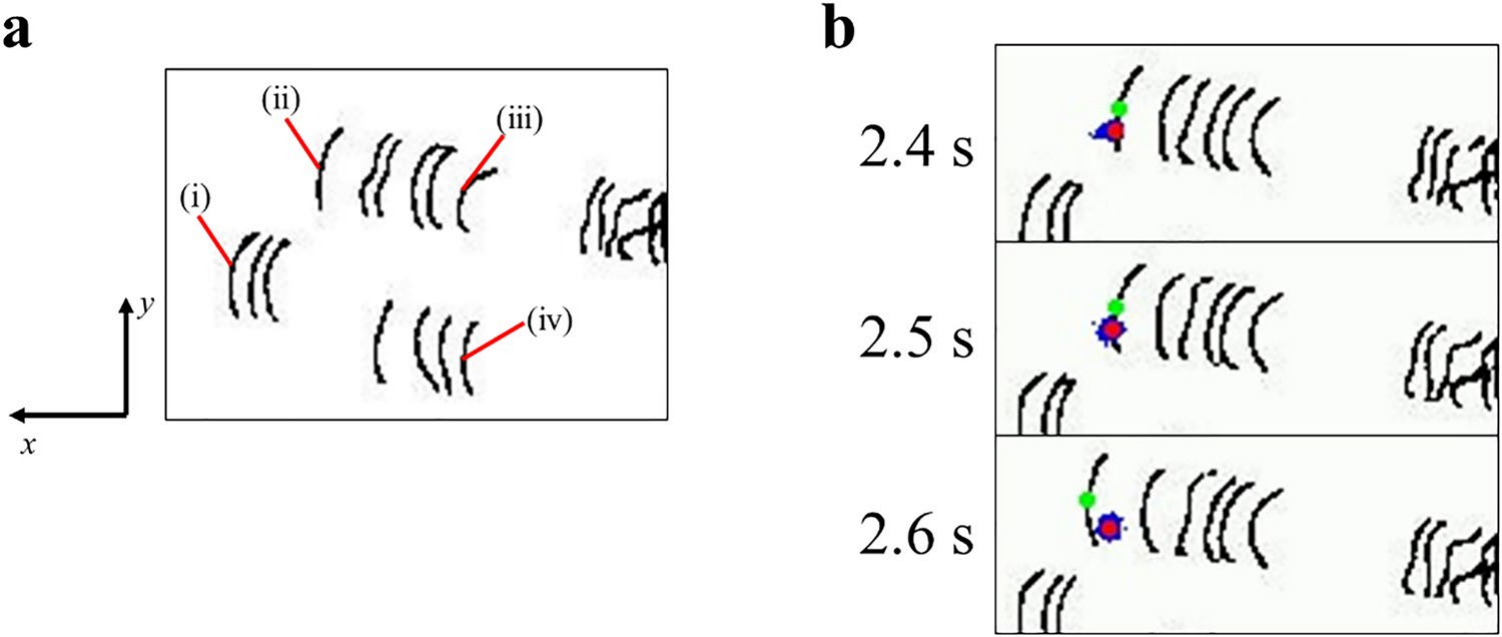
Tracking dislocations using the developed particle filter based tool. Note that this is the process of tracking the midpoints of dislocation lines using tracking indicators called “particles”, and particles are used to predict midpoints of dislocation lines as the locations of dislocations. (**a**) Four dislocations(i) ~ (iv) tracked by the tool. (**b**) Dislocation (i) tracked by particle filter. The blue dots represent the particles distributed on the field, the red dots represent the center of gravity of the blue dots, and the green dots represent the midpoints of the dislocations closest to the red dots. The green dots were treated as the location of the dislocation for measuring the velocities of dislocations.

**Figure 4:**
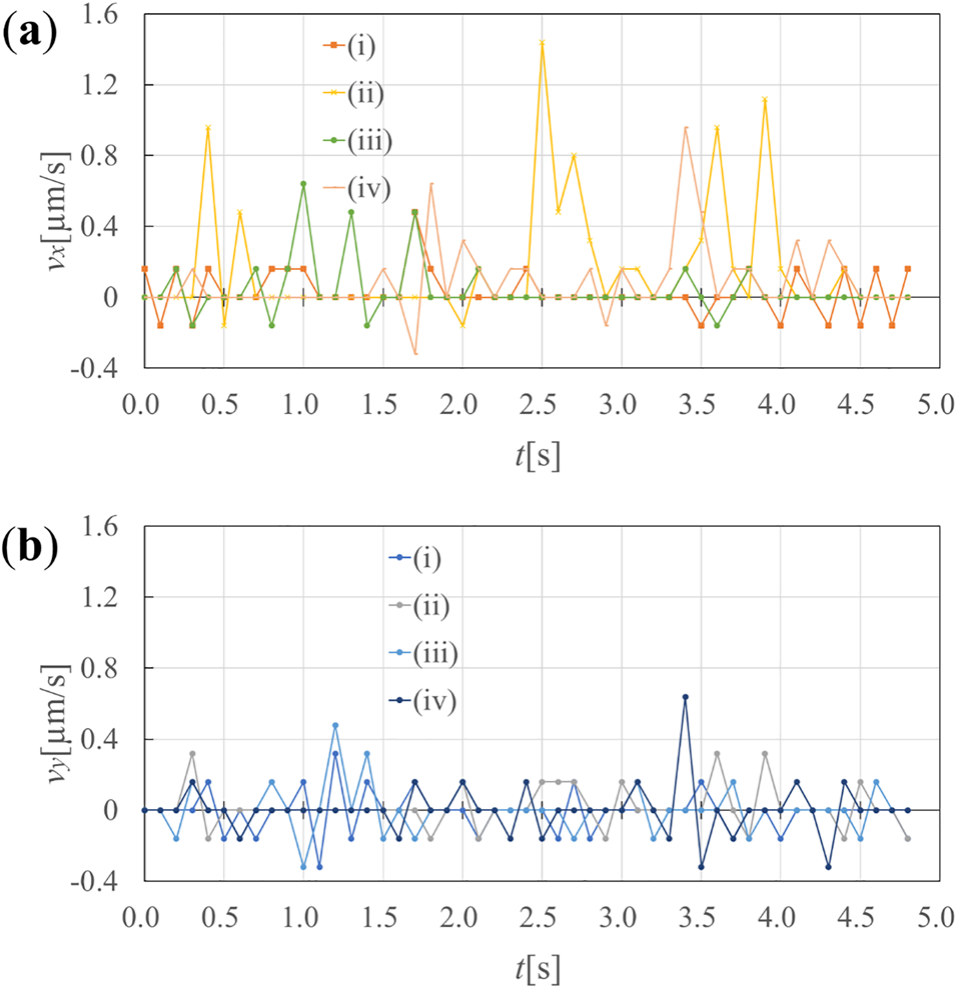
Dislocation velocity obtained from the particle filter based dislocation tracking. The dislocation tracking process is treated to be independent of the dislocation segmentation process by machine learning. (**a**) Velocity in x direction. The dislocations moved intermittently between 0–1.6 µm. (**b**) Velocity in y direction.

In here, the results of successful tracking four targeted dislocations are shown. The dislocations (i)-(iv) are shown in Fig. 3a, and the tracking of dislocation (i) is shown in Fig. 3b. In Fig. 3b, the blue dots represent the particles distributed on the field, the red dots represent the center of gravity of the blue dots, and the green dots represent the midpoints of the dislocations closest to the red dots, i.e., the coordinates of the dislocations being tracked. We confirmed that the green point stayed on a single dislocation across frames. Figure 3 shows the successful tracking of dislocations in a video in which the dislocations have been properly segmented. The stage of tracking dislocations using the particle filter is entirely independent of the stage of automatic dislocation segmentation by machine learning. Therefore, it is irrelevant whether the videos are used as a training set for machine learning. We will show the results of the dislocation velocities measured by the above tools. Figure 4 shows the velocities measured by tracking in the target directions. The average velocities of dislocations (a), (b), (c) and (d) in the ${\varvec{x}}$ direction measured by the particle filter were 0.03, 0.16, 0.04 and 0.09 µm/s, respectively, thus the average of these was 0.08 µm/s. We use this average value in the ${\varvec{x}}$ direction as the average velocity of the dislocation, because the dislocations in the TEM videos are all visually moving in the ${\varvec{x}}$ direction.

The sample used in the experiment is an austenitic steel having the face-centered cubic crystal structure thus the magnitude of the Burgers vector can be calculated as $b = 0.20 {\text{nm}}$, according to the crystal lattice of fcc iron^35^.

Since the dislocation density of the TEM image used in this study cannot be determined precisely, we calculated the dislocation density in the FOV of the TEM image and then used it as the dislocation density. The original size of the TEM video was 480 × 480 pixels, and 1 ${\text{pixel}} = 0.016\; {\mu m}$, the area of the initial FOV of the TEM video was $\left( {480\; {\text{pixel}} \times 0.016\; {\mu m}/{\text{pixel}}} \right)^{2} = 59.0\;{\mu m}^{2}$. There were 22 dislocation lines in the initial FOV of the TEM video, and by dividing the number of dislocations by the area, we obtain $\rho = 30/59.0 {\mu m}^{2} = 0.37 /{\mu m}^{2}$.

Substituting these values and the measured average dislocation velocity of 0.08 µm/s into the Eq. (1), the shear strain rate on the ($1 1 1$) plane is obtained as $\dot{\gamma } = 5.9 {\upmu }/{\text{s}}$. Here, we can find the shear strain rate in a tensile direction by dividing the shear strain rate on the ($1 1 1$) plane by the Schmid factor on the slip plane ($1 1 1$) and in the slip direction [$\overline{1} 0 1$]. We determined that the slip direction is $\left[ {\overline{1 } 0 1} \right]$ because the Schmid factor between that direction and $\left[ {\overline{1} \overline{2} 2} \right]$ is 0.136, which is the largest. Then we calculated the strain rate in the tensile direction to be 43.5 µ/s. The strain rate in the tensile direction at the experimental conditions is 100 µ/s, which is a reasonable value considering the wide range of dislocation density values.

In Fig. 4, we can observe intermittent dislocation motion. The reason for this may be that the dislocations are stationary due to localized crystal defects in the sample, which inhibit their motion, and they move when they gain an energy to overcome the obstacles and advance due to external stress. It is also possible that the elastic field from other dislocations also affect the velocity of the dislocations, as the movement of one dislocation causes the migrations of the other dislocations in the surrounding area and changes the local stress.

In Fig. 4, some plots take negative values because of some limitations and uncertainties of the method. For example, the velocity obtained by this method cannot be more accurate than the spatial resolution of experimental TEM images. This limitation will be compensated for by improving the in-situ TEM technique. In addition, position prediction using particle filters may fail if the dislocations are not sufficiently far apart from each other or if the dislocations move too rapidly.

The goal of our research is to develop a framework that can measure dislocation velocities using machine learning and a particle filter. The results of our measurements can be validated by comparing them with the results of molecular dynamics or dislocation dynamics simulation. The results can also be verified by our method itself applied to a series of experiments with various strain rates. We can qualitatively verify the results by checking whether the dislocation velocity increases with the strain rate.

Figure 4 shows the dislocation velocities in a video in which the dislocations have been segmented. The dislocation velocities are measured on the basis of the results obtained using the particle filter for dislocation tracking. The dislocation tracking process is treated as independent of the dislocation segmentation process by machine learning. Therefore, it is irrelevant whether the training set for machine learning is used for the velocity measurement.

## Discussion

In this study, we developed a Framework to detect dislocations in videos captured using TEM using U-Net and measure their migration velocity using particle filters by taking their intermittent motion and shape changes into account. The dislocation velocities were measured and confirmed to be theoretically valid, and their intermittent motions could be quantitatively evaluated.

This method has possibility to be applied not only to dislocation videos like the one used in this study, but also to videos of TEM in situ experiments (dynamic observation) on other phenomena. For example, immediate applications would be dynamically measure the velocity and analyze the shape changes of dislocations in various dislocation reactions including but not limited to Orowan mechanism (particle dispersion strengthening mechanism), grain boundary migration, and deformation twinning behavior induced by external stimuli such as magnetic field, heat or stress field. It is also possible to chase the velocity, motion and shape change of nanoparticles during an oriented-attachment reaction where dynamics in particles, translational and rotational accelerations, is critical to gain the mechanistic understanding (e.g., https://doi.org/10.1126/science.1219643).

### Time-depended evolution

Analyzing the dynamic behavior or time-dependent evolutions of nanoparticles or even single atoms and their analogues on a support material is in fact simpler than the model case we treated in here, because the projection effect is nearly negligible due to the size of the objects of interest. For example, the interparticle distance on a 2D image is not largely deviated from the true value. On the other hand, the projection effect becomes significant when analyzing nanoscale objects embedded in a media. Supplemental Fig. 2 demonstrates how the sample thickness, i.e., the length along the incident electron beam direction, develop delusions. Thus, the developed framework would prove effective when the sample thickness becomes larger and the projection effect becomes prominent, which is an emerging demand for in-situ electron microscopy where both high temporal resolution and the nanometer sized targeted objects are embedded in other materials as well as media such as liquid, or are part of a larger-scale object to observe the object’s behavior in a natural way.

## Method

**Figure 5:**
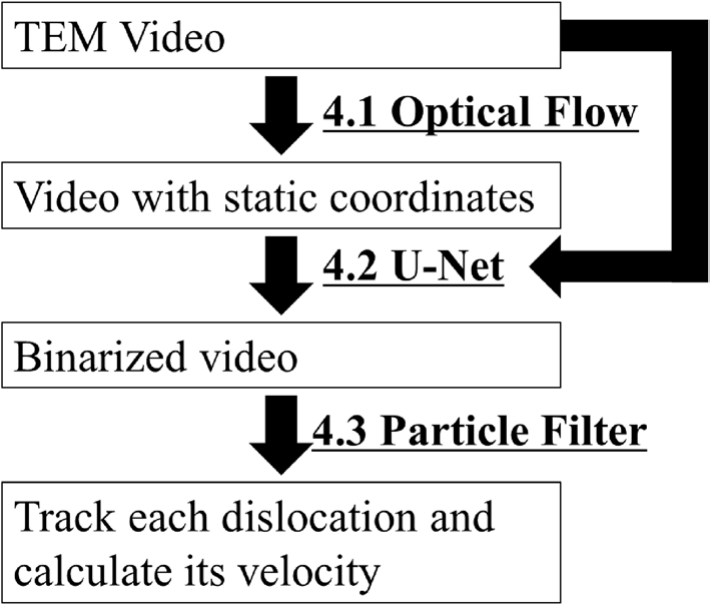
Conceptual diagram of the developed framework to assess dislocation velocities.

The configuration of the dislocation velocity measurement tool developed in this study is shown in Fig. 5. With the developed velocity measurement tool, is capable of automatic measurement of the velocity of each dislocation in the TEM video.

The framework consists of three functions: first, an optical flow to fix the field of view of the video; second, a machine learning to detect dislocations in the video; and third, a particle filter to track the locations of detected dislocations. The details are given for these three functions in the following subsections.

### Optical flow

In experimental TEM videos, we cannot accurately measure the velocity of dislocation movement because the FOV moves. Therefore, we create a static coordinate system of the TEM video by cropping a part of the frame image according to the movement of the FOV. We use optical flow^26–28^ to calculate the movement vector of the FOV between the image at time $t$ and the image at $t + \Delta t$. Considering a pixel $I\left( {x,y,t} \right)$ in an image, here, $x,y$ are the two-dimensional coordinates in the image, and $t$ is the dimension representing the time axis direction. The pixel $I\left( {x,y,t} \right)$ is supposed to have moved $\left( {\Delta x,\Delta y} \right)$ in the image after time $\Delta t$. Assuming that these two pixels are looking at the same object and that the brightness of the object in the image does not change between successive frames, the following relationship holds

2 $$I\left( {x,y,t} \right) = I\left( {x + \Delta x,y + \Delta y,t + \Delta t} \right).$$

Considering that the motion of the object is small, the Taylor expansion of the right-hand side yields

3 $$I\left( {x + \Delta x,y + \Delta y,t + \Delta t} \right) = I\left( {x,y,t} \right) + \frac{\partial I}{{\partial x}}\Delta x + \frac{\partial I}{{\partial y}}\Delta y + \frac{\partial I}{{\partial t}}\Delta t.$$

Removing the common terms from Eq. (2), we obtain

4 $$\frac{\partial I}{{\partial x}}\Delta x + \frac{\partial I}{{\partial y}}\Delta y + \frac{\partial I}{{\partial t}}\Delta t = 0.$$

Dividing both sides of Eq. (4) by $\Delta t$, the following equation is derived

5 $$I_{x} u + I_{y} v + I_{t} = 0.$$

Here,

6 $$I_{x} = \frac{\partial I}{{\partial x}}; \quad I_{y} = \frac{\partial I}{{\partial y}},$$

7 $$u = \frac{\partial x}{{\partial t}};v = \frac{\partial y}{{\partial t}}.$$

Here, $\left( {u,v} \right)$ is called the optical flow or velocity vector at pixel $\left( {x,y} \right)$. Equation (5) is called the optical flow constraint equation. The gradients of the pixels, $I_{x} , I_{y}$, and the gradient in the time axis, $I_{t}$, can be caluclated computationally. However, since there is only one constraint Eq. (5) for the two unknowns $u$ and $v$, it cannot be uniquely determined. The opensource library Open CV used in this study employes the Lucas-Kanade method^27^, which determines $u$ and $v$ by assuming that neighboring pixels move in the same way.

### Machine learning

**Figure 6:**
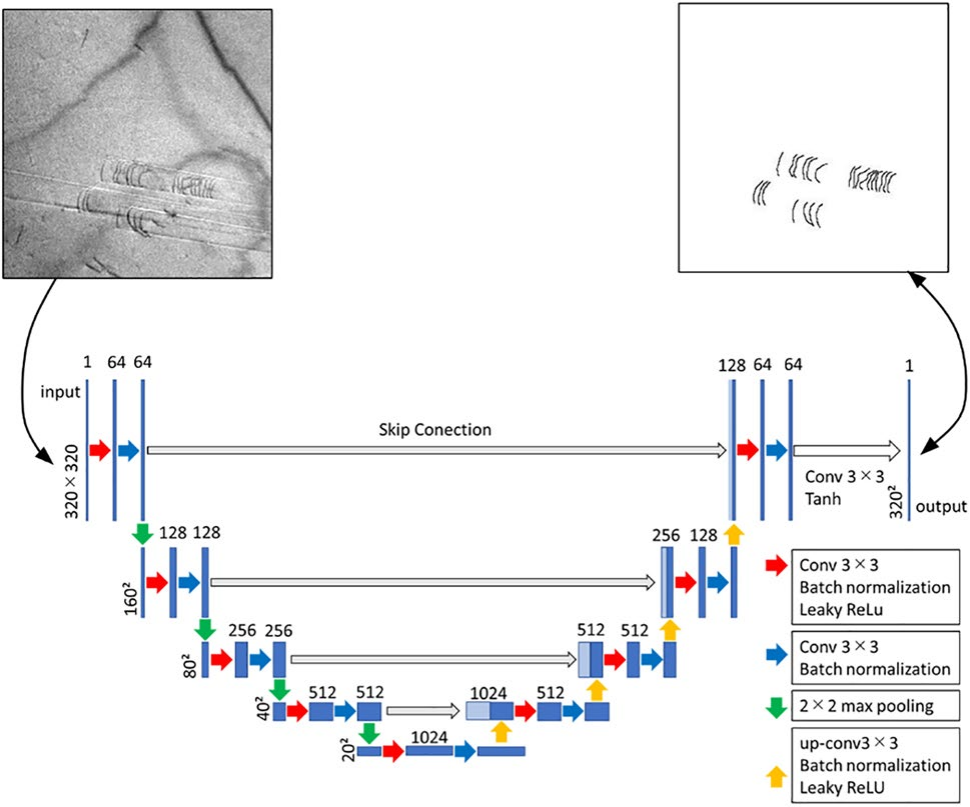
U-net architecture (for 320 × 320 pixels). Each blue square corresponds to a multichannel feature map. The light blue squares represent copied feature maps. The number of channels is shown at the top of individual boxes, and the size is shown in the lower left corner of them. Each arrow represents each operation.

We apply the machine learning model U-Net^6^, which is used for semantic segmentation, to the frame images of the experimental video to binarize the dislocations and the background. Semantic segmentation is the task of classifying an image into pixel-wise classes. U-Net consists of convolutional neural networks, with multilayer convolution, up convolution, and skip structures. U-Net has shown good results in semantic segmentation tasks. The structure of the U-Net model is shown in Fig. 6.

The applicability of the U-Net training protocol is an important, however it would be challenging to validate practically if an independent set of similar data is not available. The success of training U-net, however, can be defined as the binarization of one video based on a unified index since the ability to binarize a single video with a unified index is an important aspect of our research. In this aspect, we can say that the trained U-net successfully identifies the dislocations.

Assuming another experimental video is obtained, the current trained U-Net would be applied first to binarize it as a sanity check. It should be noted that no independent validation dataset for machine learning was used in this research because the validation dataset and test datasets were very similar to each other for the dislocation images of a TEM video.

### Particle filter

Note that this part is independent from the process of automatic dislocation segmentation by machine learning.

#### Identification of each dislocation

**Figure 7:**
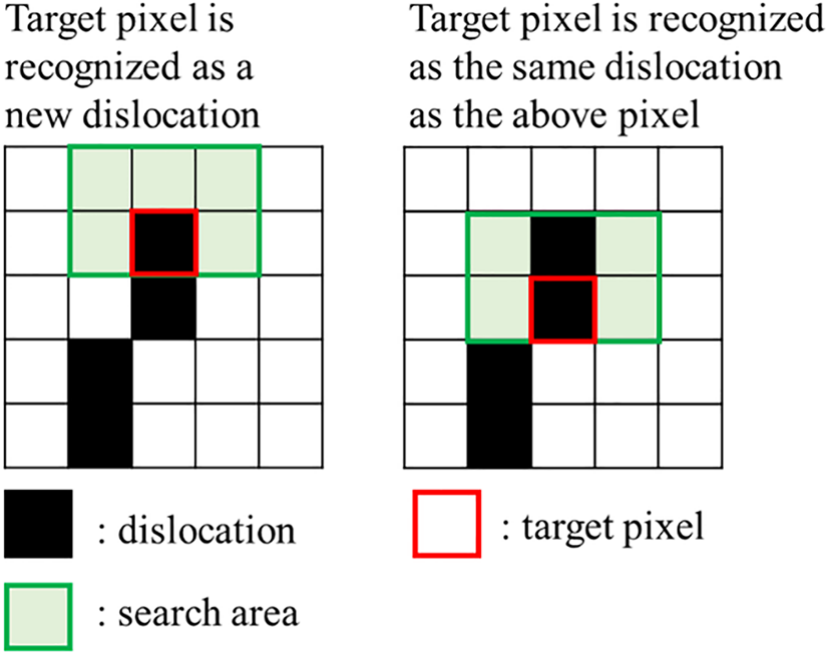
Method to identify each dislocation. This pre-process toward the particle filter determines that consecutive pixels are the same dislocation.

In the binarized image, the dislocation pixels are distinguished from the background pixels, but the dislocations are not distinguished from each other. The particle filter needs to identify the dislocations in a frame because it needs to set the target to be tracked. Therefore, we developed a program to search around the dislocation pixels and identify them as the same dislocation if they are continuous, as shown in Fig. 7.

### Particle filter to track dislocation

**Figure 8:**
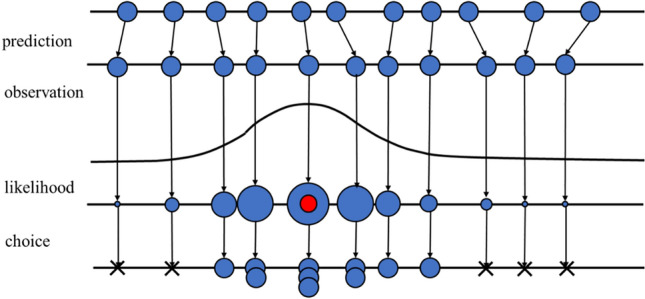
Particle filter algorithm. The particle filter enables robust tracking against noise and environmental variations.

Particle filter^33,34^ is a method for estimating the position of an object by distributing a large number of particles on the screen and using the prediction from the previous state and the current observation information. The particle filter approximates the probability distribution of the object to be tracked in the entire state space by a large number of particles with state quantities and weights (likelihoods), which enables robust tracking against noise and environmental variations. The particle filter algorithm is shown below (see Fig. 8).

1. Generate $N$ particles based on the initial coordinates of the target dislocation.
2. Move the particles based on the prediction. The prediction is based on the average velocity of all dislocations calculated by Optical Flow.
3. Obtain information necessary for likelihood calculation for each particle.
4. Calculate the likelihood for each particle based on the particle information. The likelihood is computed by the brightness of the pixel where the particle is located, and the similarity between the image of the region around each particle and the image of the region around the dislocation in the previous frame.
5. Calculate the weighted average with the likelihood of each particle as a weight.
6. Re-spread $N$ particles with a probability proportional to the high likelihood of each particle.
7. Move to the next state, and repeat from procedure 2.

By performing the above processes in each frame, particles are able to track the target object. When implementing a particle filter, it is important to design the prediction (Procedure 2) and the likelihood (Procedure 4). appropriately based on information such as the motion and shape of the target object, in order to track the target object accurately. In this study, we used the information that dislocations moved only in one direction for prediction. We also used the information that information that the pixel with the dislocation is black and the positional relationship of the dislocations does not change significantly between the previous and current frames for likelihood function.

## Supplementary Information

Supplementary Information.

## References

[1] Goodfellow I, Bengio Y, Courville A (2016). Deep Learning (MIT Press.

[2] Garcia-Garcia A, Orts-Escolano S, Oprea S, Villena-Martinez V, Martinez-Gonzalez P, Garcia-Rodriguez J (2018). A survey on deep learning techniques for image and video semantic segmentation. Appl. Soft Comput..

[3] Taghanaki SA, Abhishek K, Cohen JP, Cohen-Adad J, Hamarneh G (2021). Deep semantic segmentation of natural and medical images: A review. Artif. Intell. Rev..

[4] Garcia-Lamont F, Cervantes J, López A (2018). Lisbeth Rodriguez, Segmentation of images by color features: A survey. Neurocomputing.

[5] Treml, Michael, et al. Speeding up semantic segmentation for autonomous driving. *MLITS, NIPS Workshop*. **2**(7) 2016.

[6] Ronneberger, O., Fischer, P., & Brox, T. U-net: Convolutional networks for biomedical image segmentation. In *International Conference on Medical image computing and computer-assisted intervention*, **9351, **234-241 (2015)

[7] Skourt BA, El Hassani A, Majda A (2018). Lung CT image segmentation using deep neural networks. Procedia Comput. Sci..

[8] Liu R, Kumar A, Chen Z, Agrawal A, Sundararaghavan V, Choudhary A (2015). A predictive machine learning approach for microstructure optimization and materials design. Sci. Rep..

[9] Chowdhury A, Kautz E, Yener B, Lewis D (2016). Image driven machine learning methods for microstructure recognition. Comput. Mater. Sci..

[10] Iwasaki Y, Sawada R, Saitoh E (2021). Machine learning autonomous identification of magnetic alloys beyond the Slater-Pauling limit. Commun Mater.

[11] Stan T, Thompson ZT, Voorhees PW (2020). Optimizing convolutional neural networks to perform semantic segmentation on large materials imaging datasets: X-ray tomography and serial sectioning. Mater. Charact..

[12] Kitahara A, Holm E (2018). Microstructure cluster analysis with transfer learning and unsupervised learning. Integr Mater Manuf Innov.

[13] Zhang Y, Ngan AHW (2019). Extracting dislocation microstructures by deep learning. Int. J. Plast.

[14] Jerez D, Stuart E, Schmitt K, Guerrero-Given D, Christie JM, Kamasawa N, Smirnov MS (2021). A deep learning approach to identifying immunogold particles in electron microscopy images. Sci. Rep..

[15] Holm EA, Cohn R, Gao N, Kitahara AR, Matson TP, Lei B, Yarasi SR (2020). Overview: Computer vision and machine learning for microstructural characterization and analysis. Metall. and Mater. Trans. A..

[16] Voyles PM (2017). Informatics and data science in materials microscopy. Curr. Opin. Solid State Mater. Sci..

[17] Steinberger D, Song H, Sandfeld S (2019). Machine learning-based classification of dislocation microstructures. Front. Mater..

[18] Salmenjoki H, Alava MJ, Laurson L (2018). Machine learning plastic deformation of crystals. Nat. Commun..

[19] Kautz, E. J. Predicting material microstructure evolution via data-driven machine learning. *Patterns*, 100285 (2021). 10.1016/j.patter.2021.100285 PMC8276005 34286300

[20] Roberts G, Haile SY, Sainju R, Edwards DJ, Hutchinson B, Zhu Y (2019). Deep learning for semantic segmentation of defects in advanced STEM images of steels. Sci. Rep..

[21] Potocek P, Trampert P, Peemen M, Schoenmakers R, Dahmen T (2020). Sparse scanning electron microscopy data acquisition and deep neural networks for automated segmentation in connectomics. Microsc. Microanal..

[22] Arganda-Carreras I, Kaynig V, Rueden C, Eliceiri KW, Schindelin J, Cardona A, Sebastian Seung H (2017). Trainable Weka Segmentation: a machine learning tool for microscopy pixel classification. Bioinformatics.

[23] Johnston WG, Gilman JJ (1959). Dislocation velocities, dislocation densities, and plastic flow in lithium fluoride crystals. J. Appl. Phys..

[24] Spurgeon SR (2020). Towards data-driven next-generation transmission electron microscopy. Nat. Mater..

[25] D.Hull, D. J. Bacon, Introduction to dislocations. Mater. Sci. (2001).

[26] Horn BK, Schunck BG (1981). Determining optical flow. Artif. Intell..

[27] Lucas, et al., An iterative image registration technique with an application to stereo vision. Vancouver, British Columbia, (1991).

[28] Barron JL, Fleet DJ, Beauchemin SS (1994). Performance of optical flow techniques. Int. J. Comput. Vision.

[29] Long, J., Shelhamer, E., & Darrell, T. Fully convolutional networks for semantic segmentation. in *Proceedings of the IEEE conference on computer vision and pattern recognition, *3431–3440 (2015). 10.1109/TPAMI.2016.2572683 27244717

[30] Badrinarayanan V, Kendall A, Cipolla R (2017). Segnet: A deep convolutional encoder-decoder architecture for image segmentation. IEEE Trans. Pattern Anal. Mach. Intell..

[31] Dong, H., Yang, G., Liu, F., Mo, Y., & Guo, Y. Automatic brain tumor detection and segmentation using U-Net based fully convolutional networks. in *Annual conference on medical image understanding and analysis. *506–517 (2017).

[32] Liu Z, Cao Y, Wang Y, Wang W (2019). Computer vision-based concrete crack detection using U-net fully convolutional networks. Autom. Constr..

[33] Isard M (1998). Condensation conditional density propagation for visual tracking. Int. J. Comput. Vision.

[34] Nummiaro K, Koller-Meier E, Van Gool L (2003). An adaptive color-based particle filter. Image Vis. Comput..

[35] Alok N (1997). The Metal Databook.

