# Supplementary material for: Nanoscale defect evaluation framework combining real-time transmission electron microscopy and integrated machine learning-particle filter estimation
Source: Sci Rep. 2022 Jun 22;12:10525. doi: 10.1038/s41598-022-13878-8 (PMC9217921; doi:10.1038/s41598-022-13878-8)
Supplement: Supplementary file 1 — Supplementary Information. [file 41598_2022_13878_MOESM1_ESM.docx]

Supplemental Figures


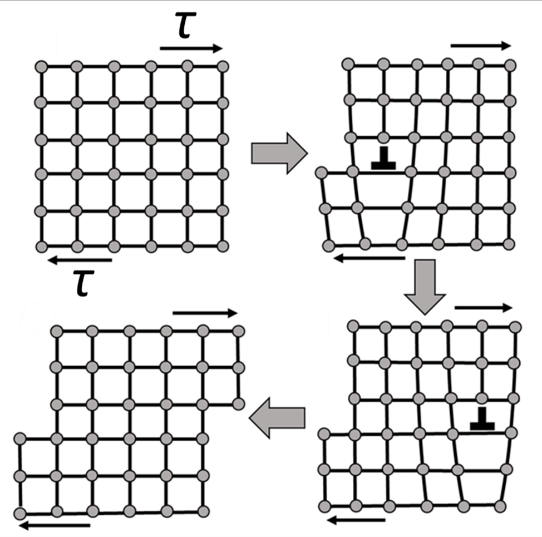


Supplemental Figure1. Slip deformation of crystals and dislocation motion.


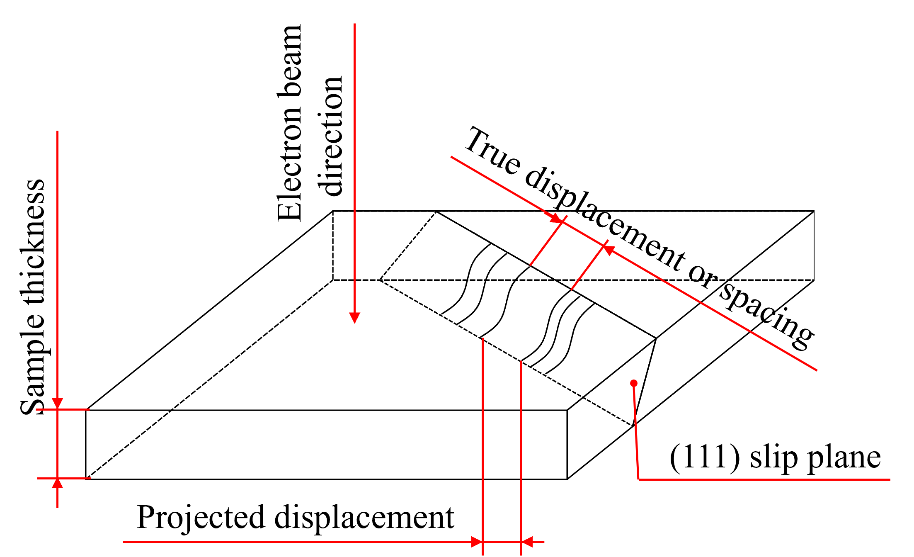


Supplemental Figure 2. The error in projecting 3D to 2D in TEM
